# Tuberculosis Meningitis in a 9-Month-Old Girl during the COVID-19 Pandemic

**DOI:** 10.1155/2024/9586953

**Published:** 2024-09-20

**Authors:** Amir-Hassan Bordbari, Kobra Sheidaee, Azin Hajialibeig, Mohammad Reza Navaeifar, Maedeh Gooran, Mohammad Sadegh Rezai

**Affiliations:** ^1^ Student Research Committee School of Medicine Mazandaran University of Medical Sciences, Sari, Iran; ^2^ Pediatric Infectious Diseases Research Center Mazandaran University of Medical Sciences, Sari, Iran

## Abstract

Tuberculous meningitis (TBM) is a serious form of TB disease that can result in high morbidity and mortality, particularly if there are delays in diagnosis and treatment. In this case report, a 9-month-old girl was admitted with persistent vomiting and focal seizures. On examination, she was found to have a right-side hemiparesis. Brain imaging showed intense nodular leptomeningeal enhancement, hydrocephalus, a hypolucent lesion in the left basal ganglia, arterial stenosis and vasculitis, and an old ischemic insult. The patient was initially diagnosed with an acute ischemic stroke and was treated with aspirin and antiepileptic drugs. The patient's condition failed to improve despite initial treatment, leading to further diagnostic procedures. The results uncovered a diagnosis of TBM. The case highlights the importance of considering TBM as a possible cause of neurological symptoms, especially during the coronavirus disease 2019 (COVID-19) pandemic where similar symptoms can be present in cases of neurological complications of severe acute respiratory syndrome coronavirus 2 (SARS-CoV-2) infection and multisystem inflammatory syndrome in children (MIS-C).

## 1. Introduction

Tuberculosis (TB) still ranks as one of the most important communicable diseases in terms of morbidity and mortality [1]. The World Health Organization (WHO) estimated that in 2021, there were 10.6 million new cases and more than 1.6 million deaths from TB. The TB incidence rate rose by 3.6% between 2020 and 2021, reversing the previous trend of approximately 2% annually declines over the past two decades [2]. In Iran, according to WHO, the overall incidence rate of TB was 11 per 100,000 in 2022 [3]. It is estimated that approximately 10% of the total TB disease burden occurs in children [4].

Miliary tuberculosis and disseminated tuberculosis are usually used interchangeably. However, the definitions are different. Miliary TB is defined as hematogenous dissemination of the bacteria resulting in widespread, caseous tubercle formations with radiologic or pathologic evidence of micronodules, while disseminated TB is defined as caseous tubercle formation in two or more extrapulmonary organs without any miliary nodular evidence [5]. Tuberculous meningitis (TBM) is the most severe form of extrapulmonary TB and causes significant morbidity and mortality, particularly if there are delays in diagnosis and treatment [6, 7]. TBM may be the only sign of TB disease in a patient [8]. The common age of occurrence of this disease following initial infection has been estimated between 6 months and 4 years [6, 9, 10].

TBM diagnosis is difficult because it often presents with nonspecific symptoms in the early stages [6, 11–13]. The clinical manifestation of TBM may be divided into three phases [9, 10, 14, 15]. In the first phase, which generally lasts for 1-2 weeks, low-grade fever, headache, irritability, drowsiness, malaise, vomiting, photophobia, listlessness, and poor weight gain/weight loss are seen. Patients in the second phase suffer from lethargy, neck rigidity, positive meningeal signs, hypertonia, seizures, vomiting, and focal neurological deficit(s). The third phase is associated with decerebrate/decorticate posturing, hemiplegia, coma, and eventually death [9, 10, 15, 16].

As severe acute respiratory syndrome coronavirus 2 (SARS-CoV-2) spreads in the world, several reports described the neurological symptoms of coronavirus disease 2019 (COVID-19) in pediatric patients. Neurological complications of SARS-CoV-2 in children include headache, seizures, encephalitis, stroke and cerebrovascular disease, Guillain–Barré syndrome, and other neuropathies. Multisystem inflammatory syndrome in children (MIS-C) is a postinfectious immune response that occurs 2-3 weeks following the COVID-19 infection. MIS-C patients can develop neurologic symptoms including seizures, headache, altered state of consciousness, and encephalopathy [17, 18].

We present a case of TBM which primarily presented with cerebral infarction and focal seizures due to brain vasculopathy. The patient was admitted at the peak of the COVID-19 pandemic. According to the patient's presentation, neurological complications of SARS-CoV-2 infection and MIS-C were suspected as well. In this case report, we discuss the similarities and differences between TBM, MIS-C, and COVID-19 neurological complications and emphasize the importance of stroke as one of the presentations of TBM.

## 2. Case Report

A previously healthy 9-month-old girl was admitted with a history of persistent vomiting for one month, right-sided hemiparesis for two weeks, and 6 episodes of focal seizures in the right limbs. She was the only child in her family, had no known past medical history, and was afebrile during the month prior to admission. She was up-to-date with all of her routine vaccines and was not in close contact with TB or COVID-19 patients.

On admission, her weight was 10 kg (90^th^–97^th^ percentile), height was 70 cm (50^th^ percentile), and head circumference was 44 cm (50^th^ percentile). Physical examination findings were significant for right-sided hemiparesis and hyperreflexia in the right limbs. However, the patient's muscle tone was normal. A Bacillus Calmette–Guérin (BCG) vaccine scar was noted on the right upper arm. Other physical examinations were unremarkable.

Her complete blood count included a white blood cell (WBC) count of 16800 cells/mm^3^ (reference value: 4 × 10^3^−11 × 10^3^/mm^3^) with a differential of 78% neutrophils, 20% lymphocytes, 1% monocytes, and 1% eosinophils; hemoglobin of 10.2 g/dL (reference value: 12–16 g/dL); and platelet count of 550 × 10^6^ cells/L (reference value: 140 × 10^3^–440 × 10^3^/mm^3^). ESR was 6 mm/hour (reference value: up to 15 mm/hour) and C-reactive protein (CRP) was 1 mg/L (reference value: 0–6 mg/dL).

**Figure 1:**
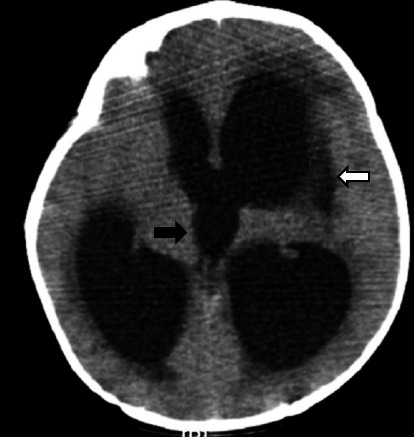
Axial brain CT during the first admission. Demonstrating moderate hydrocephalus (black arrow), with interstitial edema and encephalomalacia (destruction of brain tissue) in the left lentiform nucleus (white arrow).

**Figure 2:**
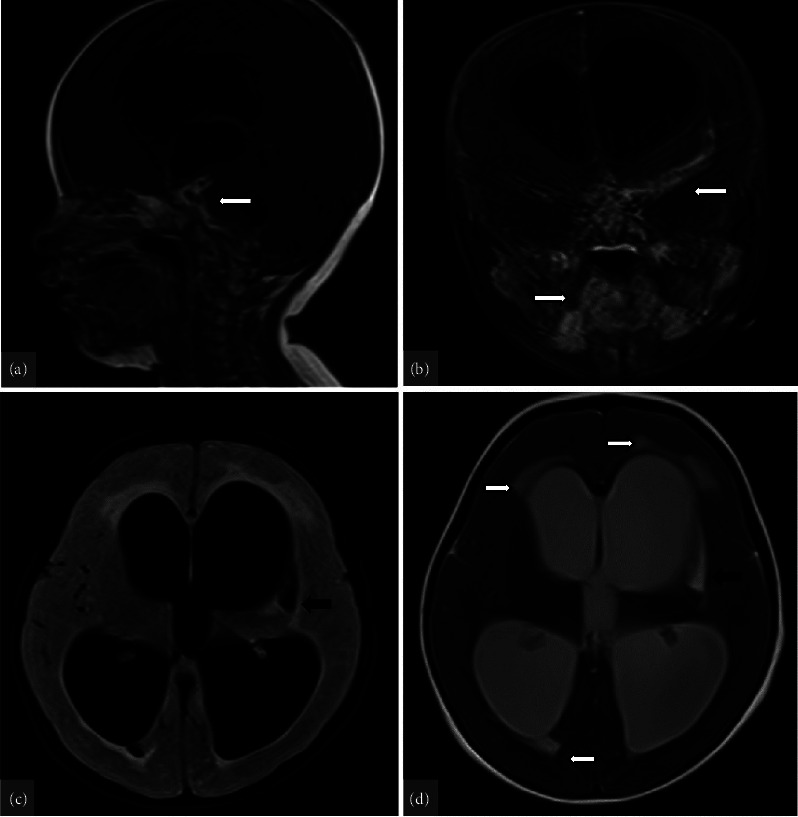
Brain MRI during the first admission. (a and b) T1 fat saturation with contrast showed intense nodular leptomeningeal enhancement in the basal, suprasellar, and prepontine cisterns and left Sylvian fissure. (c and d) FLAIR and T2W sequences showing a moderate hydrocephalus with asymmetrical lateral ventricle dilatation (right: 30 mm and left: 34 mm), leading to interstitial periventricular edema (white arrows). Black arrows show an old ischemic insult (encephalomalacia) of the left lentiform nucleus.

**Figure 3:**
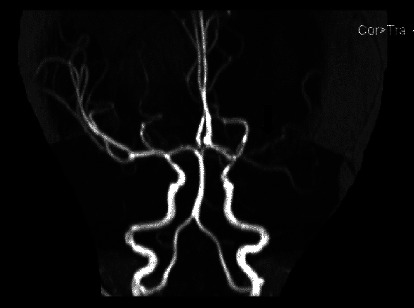
Brain MRA during the first admission. Revealing diffuse luminal narrowing of the left M1 to M4 segments of the middle cerebral artery (MCA), indicating significant arterial stenosis and vasculitis.

Brian's computerized tomography (CT) revealed hydrocephalus and a hypolucent lesion in the left basal ganglia (Figure 1). Magnetic resonance imaging (MRI) demonstrated intense nodular leptomeningeal enhancement in the basal, suprasellar, and prepontine cisterns as well as left Sylvian fissure and a moderate degree of hydrocephalus with asymmetrical lateral ventricle dilatation causing interstitial periventricular edema associated with an old ischemic insult (encephalomalacia) of the left lentiform nucleus (Figure 2). Magnetic resonance angiography (MRA) documented a diffuse luminal narrowing of the M1 to M4 segments of the left middle cerebral artery (MCA), which suggests significant arterial stenosis and vasculitis (Figure 3). Magnetic resonance venography (MRV) was normal.

Based on imaging findings, the patient was diagnosed with acute ischemic stroke (AIS). Therefore, aspirin and antiepileptic drugs were prescribed. The child was further investigated with lumbar puncture and cerebrospinal fluid (CSF) analysis showed red blood cells (RBCs) of 5/mm3, WBC of 9/mm3 with 60% polymorphonuclear neutrophil (PMN), glucose 11 mg/dL (reference value: 50–80 mg/dL), and protein 314 mg/dL (reference value: up to 150 mg/dL). Regarding the CSF analysis, an antibiotic and antiviral drug regimen including vancomycin, third-generation cephalosporin, and intravenous acyclovir was initiated. CSF culture, smear, cytology tests, and herpes simplex virus (HSV) reverse transcription polymerase chain reaction (RT-PCR) were negative, so acyclovir was discontinued. SARS-CoV-2 RT-PCR on nasopharyngeal swabs was negative. Transthoracic echocardiogram and genetic thrombophilia and other coagulation tests were normal.

Throughout the admission, the patient's symptoms gradually improved and she did not experience seizure recurrence. The patient was discharged on day 10 with aspirin, acetazolamide, and levetiracetam. Her leukocytosis disappeared and her ESR and CRP were within normal range.

**Figure 4:**
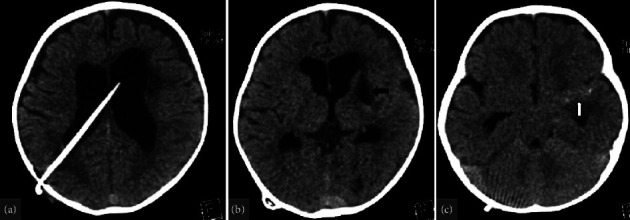
Axial brain CT scan without contrast taken during the second admission: (a) mild hydrocephalus with a ventriculoperitoneal (VP) shunt in the left lateral ventricle, (b) left lentiform nucleus encephalomalacia, and (c) arterial calcification along the left M1 segment of the middle cerebral artery (MCA).

After about 40 days, the patient returned to the emergency department with vomiting, neck stiffness, and a bulging anterior fontanelle. She was admitted with normal vital signs and a CT scan revealed a worsening in hydrocephalus. Therefore, a ventriculoperitoneal (VP) shunt placement surgery was performed to decrease the hydrocephalus level (Figure 4). The patient was intubated for surgery with synchronized intermittent mandatory ventilation (SIMV) mode with the following settings: respiratory rate (RR) 30 per minute, peak inspiratory pressure (PIP) 15 cmH_2_O, pressure support (PS) 8 cmH_2_O, positive end-expiratory pressure (PEEP) 3 cmH_2_O, and inspiratory time (TI) 0.5 seconds. Lumbar puncture (LP) was performed and the CSF analysis result was as follows: RBC of 600/mm3, WBC of 220/mm3 with 95% PMN and 5% lymphocytes, glucose 17 mg/dL, and protein 207 mg/dl.

Regarding the hydrocephalus, TB was suspected and a tuberculin skin test (TST) or a purified-protein derivative (PPD) skin test via the Mantoux technique was positive (>20 mm). Therefore, a standard four-drug regimen for tuberculosis treatment (isoniazid, rifampin, ethambutol, and pyrazinamide) was initiated. However, drug susceptibility testing was not possible as the TB PCR test and TB culture results were negative. The plan was to continue this four-drug regimen for two months and then maintain the use of isoniazid and rifampin for an additional 10 months. In addition, 10 mg of vitamin B6 was started once a day. Moreover, SARS-CoV-2 serology (IgG and IgM) on blood, CSF Ziehl–Neelsen stain, Mycobacterium tuberculosis PCR, and mycobacterial culture were all negative. Chest radiography was normal.

The patient stayed in the PICU for 38 days. Through admission, several ventricular and lumbar CSF analyses were performed and the results showed that the patient was gradually improving (Table 1). The patient was discharged on day 41 in good general condition.

**Figure 5:**
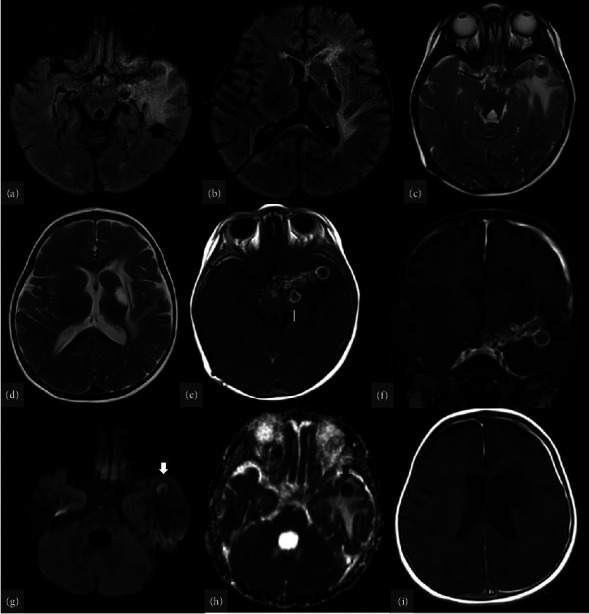
Follow-up MRI with various sequences. T2W/FLAIR sequences (a–d) reveal vasogenic edema in the left frontotemporal lobes, causing ventricular compression. Some round lesions are also visible, which appear hyperintense on T1W and isointense to the cortex on T2W. Following contrast administration (e, f) leptomeningeal enhancement and rim-enhancing nodules appear in the prepontine cistern and perisylvian fissure. Central diffusion restriction is seen on DWI/ADC (g, h), along with thickened pachymeningeal enhancement in the left frontoparietal region (i).

During follow-up visits, the patient exhibited appropriate physical growth and age-appropriate mental development, remaining free from seizure episodes, hemiparesis, and neurological symptoms. In the ophthalmologic examination, bilateral mild optic nerve atrophy was detected. Follow-up CT and MRI four months after the initiation of TB treatment showed no recurrence of hydrocephalus. However, after contrast administration, abnormal leptomeningeal enhancement with multiple rim-enhancing nodules was seen at the prepontine cistern and perisylvian fissure that was suggestive of caseating tuberculomas (Figure 5). Therefore, the two-drug regimen (isoniazid and rifampin) for TB treatment was maintained for a total of 22 months. Subsequent follow-up imaging revealed a reduction in the number and size of tuberculomas.

## 3. Discussion

We report a 9-month-old girl who was admitted to our hospital twice with hydrocephalus and tuberculous meningitis (TBM). In the first admission, the patient presented with persistent vomiting, right-sided hemiparesis, and several episodes of focal seizures. The brain imaging showed intense nodular leptomeningeal enhancement, hydrocephalus, a hypolucent lesion in the left basal ganglia, arterial stenosis and vasculitis, and an old ischemic insult. Empirical antibiotics and antivirals were also started based on CSF analysis. 40 days after discharge, she was hospitalized for the second time with symptoms of vomiting, neck stiffness, and bulging anterior fontanel. Imaging showed that the hydrocephalus had worsened and a ventriculoperitoneal (VP) shunt was inserted. The patient's deteriorating condition and hydrocephalus prompted further diagnostic tests, leading to the diagnosis of TBM.

As our patient was admitted during the peak of the COVID-19 pandemic, it was initially suspected that her stroke could be linked to novel conditions such as neurological complications of SARS-CoV-2 infection and MIS-C [17, 18]. Instances of stroke in children with COVID-19 have been documented in various case reports. Mirzaee et al. reported a 12-year-old boy who presented with ischemic stroke with symptoms of seizures, right hemiparesis, and dysarthria [19]. In imaging, unilateral focal vasculopathy, characterized by focal irregular narrowing and banding of the proximal left middle cerebral artery, was found. In this case, SARS-CoV-2 evidence was observed in nasal swabs and CSF, but CSF analysis was within normal range. In our case, no evidence of infection with COVID-19 was found. However, the worsening 4-chamber hydrocephalus and abnormal CSF analysis with no other explanation led us to a diagnosis of TBM.

Another case was reported by Tiwari et al., involving a 9-year-old girl diagnosed with MIS-C [20]. This patient was admitted with fever, headache, vomiting and 7th cranial nerve palsy, hemiplegia, brisk deep tendon reflexes, and extensor plantar response on the right. Imaging evidence was in favor of stenosis in cerebral arteries and ischemic stroke. CSF analysis documented a WBC of 50/mm3 with 80% lymphocytes, glucose 45 mg/dL, and protein 60.1 mg/d. Although our case had neurological symptoms similar to Tiwari's case, she did not meet the criteria for MIS-C. Another difference between the two cases was the CSF analysis. In our case, several lumbar and ventricular CSF tests were performed throughout the admission, which showed abnormal results (Table 1).

While CNS infections were promptly investigated upon first admission, the diagnosis of TBM was delayed. The delayed diagnosis of TBM in a child presenting with stroke has been documented in previous case reports. Radmanesh et al. reported a delayed diagnosis of a TBM patient who was presented with acute ischemic stroke [1]. During a surgical procedure, they found a peritoneal thickening at the distal end of a VP shunt. Later, peritoneal biopsy along with CSF PCR confirmed TB diagnosis. In our case, the patient originates from an endemic TB region and exhibits notable clinical features including 4-chamber hydrocephalus, ischemic stroke, and MCA narrowing. Despite negative results from various tests such as the CSF Ziehl–Neelsen stain, Mycobacterium tuberculosis PCR, and mycobacterial culture, clinical indicators, coupled with a positive tuberculin skin test, ultimately confirmed the diagnosis of TBM [13].

As demonstrated here, the clinical and imaging features of TBM may be misdiagnosed with other infectious and noninfectious etiologies such as COVID-19 and MIS-C. In this case, a broad differential diagnosis was initially considered including neurological complications of SARS-CoV-2 infection, MIS-C, vasculitis, thrombophilia, and CNS infections. However, this diagnostic approach, inadvertently led to a delay in diagnosing TBM. This case report reminds us that when an infant residing in an endemic TB region presents with 4-chamber hydrocephalus and ischemic stroke, and lacks indications of MIS-C or COVID-19, physicians should consider TB and conduct prompt diagnostic tests to prevent delays in both diagnosis and treatment.

In Iran, as a TB-endemic country, Bacillus Calmette–Guérin (BCG) vaccination is routinely given to all newborn babies. BCG vaccination protects children from severe forms of TB and high rates of mortality [14, 16]. In our case, despite BCG vaccination, the patients suffer from TBM which is a severe form of TB.

The COVID-19 pandemic caused a slowdown, interruption, or even reversal in the progress made in combating tuberculosis (TB) up until 2019, leading to an increase in TB incidence and mortality [2]. Global studies have reported delays in TB diagnosis during the pandemic, driven by disruptions in healthcare services, reduced access to medical facilities, and the reallocation of resources to combat COVID-19 [21]. Consequently, it is likely that the pandemic also contributed to an increase in the severity of TB in patients at the time of diagnosis and treatment [22]. Although it cannot be determined with certainty, in the case we reported, these factors should be considered as contributing influences.

## 4. Conclusion

In conclusion, we report an infant from a TB-endemic region with hydrocephalus, ischemic stroke, MCA narrowing, and focal seizure episodes, diagnosed with tuberculosis meningitis. Clinicians should consider TBM in similar cases to avoid misdiagnosis, especially in the absence of COVID-19 or MIS-C indications.

## Figures and Tables

**Table 1:** Lumbar and ventricular CSF analyses results during the second admission.

Day of admission	Glucose (mg/dl)	Proteins (mg/dl)	WBC count	WBC differential	RBC count
Day 1 (lumbar)	17	207	220	Neutrophil: 5%, lymphocyte: 95%	600
Day 1 (ventricular)	22	200	0	N/A	5
Day 3 (ventricular)	22	35	40	Neutrophil: 40%, lymphocyte: 60%	800
Day 7 (ventricular)	16	132	10	Neutrophil: 0%, lymphocyte: 100%	400
Day 10 (ventricular)	14	147	10	Neutrophil: 10%, lymphocyte: 90%	320
Day 12 (ventricular)	25	145	20	Neutrophil: 0%, lymphocyte: 100%	300
Day 19 (lumbar)	29	88	300	Neutrophil: 0%, lymphocyte: 100%	1400
Day 20 (ventricular)	35	145	3	N/A	380
Day 31 (ventricular)	31	47	2	N/A	1600

WBC, white blood cells; RBC, red blood cells; N/A, not available.

## Data Availability

The data used to support the findings of this study are available from the corresponding author upon reasonable request.
